# Relief of diabetes by duodenal-jejunal bypass sleeve implantation in the high-fat diet and streptozotocin-induced diabetic rat model is associated with an increase in GLP-1 levels and the number of GLP-1-positive cells

**DOI:** 10.3892/etm.2015.2669

**Published:** 2015-08-04

**Authors:** JINQUAN SHUANG, YING ZHANG, LIMEI MA, XUEMING TAN, JING HUANG, XIANG WANG, GUANYIN XIONG, ZHONGHUA JIANG, XIUHUA ZHANG, SHIQING DU, YONGSONG GU, XIANGYANG SHI, ZHINING FAN

**Affiliations:** 1 Department of Digestive Endoscopy and Medical Center for Digestive Diseases, Second Affiliated Hospital of Nanjing Medical University, Nanjing, Jiangsu 210011, P.R. China; 2 Department of Gastroenterology, People's Hospital of Chizhou, Chizhou, Anhui 247100, P.R. China

**Keywords:** duodenal-jejunal bypass sleeve, diabetes mellitus, streptozotocin, rat model, glucagon-like peptide-1

## Abstract

A recently invented duodenal-jejunal bypass sleeve (DJBS) implanted in the duodenum and proximal jejunum has exhibited good glycemic control in diabetes mellitus. However, the specific mechanism by which DJBS placement induces the remission of diabetes is not well known. Previous studies have indicated that changes in the pattern of gut hormone secretion may play a role. The aim of the present study was to explore the role of intestinal L cells and the production of glucagon-like peptide-1 (GLP-1) by these cells in DJBS implantation-induced glycemic control in diabetic rats. A DJBS was placed in the proximal small intestine of rats with diabetes induced by a high-fat diet and low-dose streptozotocin (STZ), and the effects of the DJBS on the remission of diabetes and the GLP-1 levels of plasma and intestinal tissues were investigated 12 weeks after DJBS placement. The number of intestinal GLP-1 positive cells was also counted. When the DJBS had been in place for 12 weeks, the plasma glucose level of the DJBS-implanted rats decreased significantly from 23.33±1.56 mmol/l prior to surgery to 7.70±0.84 mmol/l and the diabetes mellitus was relieved completely; however, diabetic control rats and diabetic rats subjected to sham surgery did not show any improvement. Parallel with the remission of diabetes, the plasma and distal ileum GLP-1 levels of rats in the DJBS implantation group were also higher than those of rats in the diabetic control and sham surgery groups. The number of GLP-1-positive cells in the distal ileum was also higher in the DJBS implantation group than in the diabetic control and sham surgery groups (31.0±2.6 vs. 23.5±4.4 vs. 23.0±3.2 respectively; P<0.01). DJBS implantation effectively led to the remission of diabetes in rats with diabetes induced by a high-fat diet and low-dose STZ when implanted for 12 weeks. The remission of diabetes may be associated with the increase in the number of L cells and elevation of GLP-1 levels induced by DJBS implantation.

## Introduction

The incidence of diabetes mellitus is increasing at an alarming rate in developed and developing countries (1,2). Diet, exercise and medications remain the primary choice of type 2 diabetes mellitus (T2DM) therapy, but the long-term success rate of lifestyle modifications can be disappointing. Despite the emergence of new antidiabetic drugs, the glycemic control achieved is far from perfect and some drugs may cause weight gain and increase the risk of cardiovascular disease (3–5). However, in cases where behavioral and pharmacological strategies prove insufficient in the treatment of T2DM, several types of gastrointestinal surgery can provide effective alternatives. Roux-en-Y gastric bypass (RYGB) surgery is one of the most effective and commonly performed procedures worldwide (6). Following RYGB surgery, up to 40–85% patients achieve complete remission from T2DM, and lifelong T2DM remission has been reported amongst those patients (7–9). Regardless of the good therapeutic effect that bariatric surgery has on T2DM, bariatric surgery can have complications and its total mortality rate is 0.28% [95% confidence interval (CI), 0.22–0.34] within 30 days after surgery, and 0.35% between 30 days and 2 years (95% CI, 0.12–0.58) after surgery (10). The proportion of patients seeking to have bariatric surgery is <1% (11). However, the implantation of a recently invented duodenal-jejunal bypass sleeve (DJBS) in the duodenum and proximal jejunum can mimic the biliopancreatic diversion effect of RYGB surgery and has comparable ability to induce weight loss and glycemic control with minimal invasion (12).

The weightloss-inducing effect of DJBS implantation was first verified by Milone *et al* (13) in a porcine model. Gersin *et al* (14) placed the first DJBS with the aid of gastroscope in a morbidly obese patient for 3 months. The patient achieved a total weight loss of 9.09 kg without severe complications. A series of clinical trials confirmed the excellent therapeutic effect of DJBS on diabetes mellitus (15,16). However, the specific mechanism by which DJBS placement induces glycemic control in obese and diabetic patients is not well known. Studies have found that gut hormone secreted from enteroendocrine cells plays an important role in bariatric surgery- and DJBS-mediated glycemic control (17,18), and that glucagon-like peptide-1 (GLP-1) produced by intestinal L cells is one of the most important gut hormones.

The gastrointestinal tract is important in metabolic diseases, including diabetes mellitus (19). The plasma insulin response to intravenous glucose is only 30–40% of that to oral glucose (20), that is, orally ingested glucose triggers nearly 2-3-fold more insulin secretion than the same amount of glucose delivered intravenously. The involvement of intestinal hormones in the ‘incretin effect’ has been described in the classical concept of the ‘entero-insular axis’ (21,22). Two hormones, glucose-dependent insulinotropic polypeptide (GIP) and GLP-1, take important roles in this phenomenon. The combined action of GLP-1 and GIP is believed to account for up to 70% of the total insulin secretory response after a meal (23). GLP-1 is mainly expressed in mucosal L cells located predominantly in the distal intestine (ileum and colon). In humans, a large number of GLP-1-expressing cells are distributed in the distal jejunum and ileum, the cell density increases from the proximal to the distal colon and the highest number is in the rectum, whereas in rat, the highest cell density is in the ileum (24).

GLP-1 is secreted from L cells located in the intestine and has a significant effect on glycemic control. In the rat RYGB model, studies have found that duodenal jejunum bypass increases the plasma GLP-1 level and augments the total intestinal L-cell number (25); similar increases in GLP-1 level have also been observed in diet-induced obese rats (26) and diabetic patients (16) after DJBS implantation. However, it is not clear whether the increase in circulating GLP-1 levels after DJBS implantation is caused by enhanced GLP-1 synthesis, or an increase in the intestinal L-cell number. Intestinal L-cell and mucosal hypertrophy following RYGB surgery has been reported in diabetic rats (25). Since DJBS mimics most of the effects of RYGB surgery, the present study aimed to observe the effects of DJBS implantation on glycemic control, plasma GLP-1 levels and L-cell numbers in diabetic rats, and thereby to explore the role of intestinal L cells and their production in the DJBS implantation-mediated remission of diabetes.

## Materials and methods

### 

#### Materials

Streptozotocin (STZ) was purchased from Sigma-Aldrich (St. Louis, MO, USA) and stored at −20°C in the dark. STZ was dissolved in citric acid solution (pH 4.2–4.5) prior to injection. Insulin was purchased from Tonghua Dongbao Pharmaceutical Co., Ltd. (Jilin, China). Chloral hydrate was purchased from Sigma-Aldrich and dissolved in 0.9% NaCl solution to provide a final concentration of 10% prior to use. Dipeptidyl peptidase-4 (DPPIV) inhibitor was purchased from Santa Cruz Biotechnology (Dallas, TX, USA) and dissolved in DMSO to provide a final concentration of 100 mM.

#### Animals and diet

In total, 40 Sprague-Dawley male rats weighing 200±10 g were obtained from Qinglongshan Experimental Animal Center (Nanjing, China; permission no: SOXKLSU 2009-0001). The rats were housed in a temperature- (22±2°C) and humidity (55±5%)-controlled room and kept on a 12:12 light-dark cycle (light on at 06:00 a.m.). Rats were kept in standard polypropylene cages (two rats/cage); food and water were provided *ad libitum* unless otherwise indicated. The study was also approved by the Animal Use and Care Committee of Nanjing Medical University (Nanjing, China).

#### Development of type 2 diabetes in rats with a high-fat diet and low-dose of STZ

After acclimating for 7 days, 8 rats were randomly selected from the 40 rats and allocated to the normal diet control group (Ncontrol, n=8) to receive standard rat chow (Qinglongshan Experimental Animal Center) throughout the study. The other 32 rats had access to a high-fat diet (containing 60% energy from fat; Trophic Animal Feed High-Tech Co., Ltd., Nantong, China) *ad libitum* to induce insulin resistance. All rats had free access to the assigned diet and tap water for 8 weeks continuously. Rat body weight was measured every 2 weeks, and fasting glucose was tested every 4 weeks using a hand glucometer (One Touch Ultra; Lifescan, Johnson & Johnson, Chesterbrook, PA, USA). At week 8, all rats received an oral glucose tolerance test (OGTT) and insulin tolerance test (ITT); rats with impaired OGTT and ITT were selected to receive a low-dose intraperitoneal injection of STZ (35 mg/kg) after overnight fasting. Citrate buffer (vehicle) alone was injected into control rats; normal diet-fed rats also received same dose of STZ. One week after the STZ injection, rats with non-fasting plasma glucose ≥16.7 mmol/l were considered diabetic (27) and randomized into three groups according to the plasma glucose level: Diabetes mellitus control (DMcontrol, n=8); diabetes mellitus sham surgery (DMsham, n=8), which underwent a sham surgery; and diabetes mellitus with DJBS implantation group (DMdjbs, n=8). Following the DJBS placement surgery, all diabetic rats were fed with normal standard rat chow until the end of the experiment. At 12 weeks after DJBS implantation, all rats were sacrificed with 10% chloral hydrate (Nanjing Shenbeijia Biotechnology Co, Ltd., Nanjing, China) (2 ml/100 g body weight) and intestinal tissues were collected and fixed in 4% formaldehyde for paraffin embedding or tissue homogenation.

#### DJBS implantation and sham surgery

**Figure 1.**
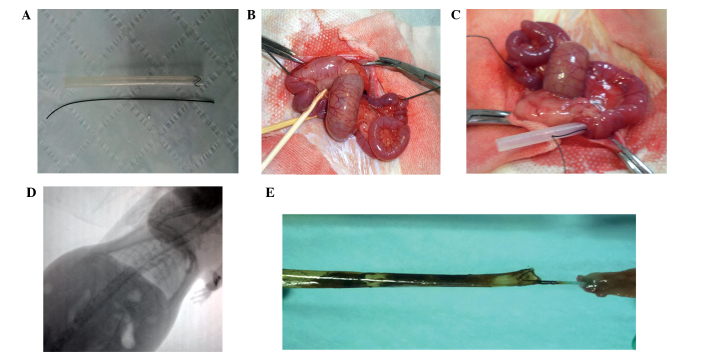
(A) A guide-wire and duodenal-jejunal bypass sleeve (DJBS) used in the study. (B) The guide-wire was introduced from the proximal jejunum and left in the intestinal lumen. (C) The DJBS was released in the duodenum and proximal jejunum. (D) The proximal metal crown was visible in an X-ray. (E) The metal crown of the sleeve remained tightly sutured to the gastric antrum, and the sleeve remained intact without any breakage.

The DJBS was made by Garson Medical Stent Apparatus Co., Ltd. (Changzhou, China) with reference to the tubes used by Aguirre *et al* (26). The DJBS is a nutrient-impermeable, flexible tube with a self-expandable metal anchor crown at the proximal end. The length and diameter of the tube are 10 cm and 5.5 mm, respectively (Fig. 1A). After release, the metal crown of the tube anchors to the rat duodenal bulb and the soft sleeve unfolds completely to the proximal jejunum below the Trietz ligament. Thus chyme from the stomach can be delivered directly to the proximal jejunum without contacting the mucosa of the proximal intestine, and the mixing of chyme with bile and pancreatic juice is also delayed until the proximal jejunum.

After overnight fasting, rats were anesthetized by the administration of 10% chloral hydrate at a dose of 3 ml/kg body weight. A midline laparotomy was made and the proximal intestine was released from the ligament of Trietz. A 5-mm-long incision was made in the front wall of the gastric antrum, and then a 0.035-inch hydrophilic guide-wire with a soft tip was smoothly inserted from the incision without injuring the enteron until it was 2 cm below the Trietz ligament. When the tip of the guide-wire was 2 cm below the Trietz ligament, the jejunum was punctured by a needle just opposite to the tip of the guide-wire, and the guide-wire was pulled from the puncture hole (Fig. 1B). The distal end of the DJBS was then sutured to the other tip of the guide-wire by a silk thread and the DJBS was pulled into the intestinal lumen by withdrawal of the guide-wire and the thread. When the sleeve was completely unfolded, the silk thread was cut near to the sleeve without pulling the sleeve out of the intestinal wall (Fig. 1C). The two incisions were then repaired, and the crown of the DJBS was moved to the duodenum bulb and sutured to the gastric antrum wall with a nylon thread. Finally, all incisions were closed by thread suture. The sham surgery consisted of laparotomy with release of the proximal intestine from the ligament of Trietz, and also gastric antrum incision, guide-wire insertion and jejunum puncture, but did not include the DJBS placement. The duration of anesthesia was standardized to 1 h for both surgical groups. After surgery, rats were fasted for 1 day and then fed a liquid diet for 2 days before being transferred to normal chow. Starting during post-operative week 2 (pow2), animals were evaluated by fluoroscopy weekly to ensure that the DJBS remained in place (Fig. 1D). The position of the DJBS and that the sleeve film remained intact were eventually verified by necropsy (Fig. 1E) after the rats were sacrificed. The non-fasting blood glucose levels and body weights of the rats were tested every 2 weeks after surgery.

#### OGTT

After overnight fasting, the OGTT test was performed. A 50% glucose solution gavage (1 g/kg) was administered and glucose levels in a blood sample taken from the tail vein were measured using a glucometer (One Touch Ultra) at 0, 30, 60, 120 and 180 min after glucose administration. The area under the curve (AUC) of glucose was calculated using the trapezoidal method.

#### ITT

After an overnight fasting, a dose of 0.4 IU/kg human insulin (Tonghua Dongbao Pharmaceutical Co., Ltd., China) was injected intraperitoneally into conscious rats. Blood glucose levels were measured using a glucometer (One Touch Ultra) at baseline and 30, 60, 90 and 120 min after insulin injection.

#### Plasma GLP-1 analysis

Prior to DJBS implantation (pow0) and at 12 weeks after surgery (pow12), after an overnight fasting, all rats received a glucose gavage (2 g/kg). At 20 min after glucose administration, blood samples were collected from the tail vein of all rats into an EDTA tube containing DPPIV inhibitor IV, K579 (cat. no. sc-202583; Santa Cruz Biotechnology). Blood samples were centrifuged at 1,640 × g for 10 min and isolated plasma was stored at −80°C for further detection. Plasma total GLP-1 levels were tested with enzyme-linked immunosorbent assay (ELISA) kits specific to rat GLP-1 (Santa Cruz Biotechnology) according to the manufacturer's instructions.

#### Intestinal GLP-1 analysis

At 12 weeks after DJBS implantation, rat intestinal tissue (proximal jejunum, distal ileum, middle colon and rectum) were sampled under anesthesia for tissue homogenization. The intestinal tissue supernatant was produced as described by Liu *et al* (28). The GLP-1 level of the intestinal tissue supernatant was tested by the same GLP-1 enzyme-linked immunosorbent assay kits as were used for plasma analysis, according to the manufacturer's instructions.

#### Immunohistochemical staining and GLP-1-positive cell count in intestinal tissues

Tissues from the proximal jejunum, distal ileum, middle colon and rectum tissues were collected at 12 weeks after the surgery. The tissues were fixed in 4% formalin for 24 h at 4°C and then embedded in paraffin blocks. Serial sections (5 µm) were cut from the paraffinized tissues. After dewaxing and rehydration, antigen retrieval was conducted at 95°C with 1X phosphate-buffered saline (PBS) buffer (pH 7.4) for 10 min using a microwave (Galanz, Guangdong, China). Immunohistochemical staining was performed using mouse monoclonal anti-GLP-1 (cat. no. sc-57166; Santa Cruz Biotechnology: 1:50). The UltraVision Quanto Detection System HRP (Thermo Fisher Scientific, Inc., Waltham, MA, USA) was used according to the manufacturer's instructions. After immunohistochemical staining, sections were dehydrated through graded ethanol and covered with a glass coverslip prior to vitrification with xylene.

The number of GLP-1-positive cells was counted in three non-serial cross-sections of proximal jejunum, distal ileum, middle colon and rectum from each rat. Specific immunoreactive cells were observed under a conventional light microscope[CX31; Olympus (China) Co., Ltd., Beijing, China] and photomicrographs were taken with a digital camera (Olympus Corporation, Tokyo, Japan; final magnification, x200). The number of GLP-1-positive cells was counted in 10 randomly chosen x200 fields in each section for all animals, Only cells with definite nuclei were counted. All values were presented as the mean ± standard deviation (SD). The GLP-1 positive cell number of each section was expressed as the number of GLP-1 positive cells per high-powered field (HP).

#### Statistical analysis

Data are presented as means ± SD. Data were analyzed by repeated measures, analysis of variance (ANOVA) or multivariate ANOVA (MANOVA), as appropriate. Glucose and insulin tolerance tests were evaluated by AUC analysis. Pairwise comparisons between different groups at different time points were analyzed by least significant difference. P<0.05 was considered to indicate a statistically significant difference. Statistical analysis was conducted using the commercially available SPSS software package, version 18.0 (SPSS, Inc., Chicago, IL, USA). Graphs were drawn using Prism (GraphPad, San Diego, CA, USA).

## Results

### 

#### Effect of a high-fat diet on the glucose and insulin tolerance of rats

**Figure 2.**
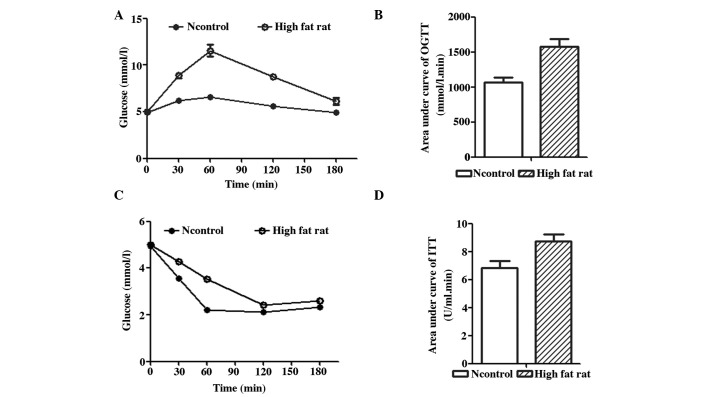
OGTT and ITT results. (A) OGTT test results of rats after feeding with a high-fat diet for 8 weeks. (B) The area under the curve of the rats fed a high-fat diet was significantly higher than that rats fed with a normal diet (F=62.56; P<0.01). In the ITT test, (C) the plasma glucose levels of the rats fed a high-fat diet decreased more slowly than those of the rats fed with a normal diet fed rats after insulin injection; (D) the area under the curve of the rats fed a high-fat diet was also higher than that of the rats fed with a normal diet (F=28.68; P<0.01). OGTT, oral glucose tolerance test; ITT, insulin tolerance test; Ncontrol, normal control.

As shown in Fig. 2, after being fed a high-fat diet for 8 weeks, rats exhibited insulin resistance as evidenced by impaired oral glucose tolerance and insulin tolerance (Fig. 2A and C). In the OGTT, the plasma glucose level of the rats fed a high-fat diet increased quickly and then decreased slowly (Fig. 1A). The AUC in the OGTT of rats fed a high-fat diet (Fig. 2B) was signiﬁcantly higher than that of rats fed a normal diet (1,576.50±106.24 mmol/L.min vs. 1,066.75±72.98 mmol/L.min; F=62.56; P<0.01). In the ITT, the AUC of rats fed a high-fat diet (Fig. 2D) also increased significantly compared with that of rats fed a normal diet (8.74±0.49 vs. 6.83±0.52 U/ml.min; F=28.68; P<0.01). The impairment observed in the OGTT and ITT indicated that the rats fed a high-fat diet were insulin resistant.

#### Characteristics of the rats with diabetes induced by a high-fat diet and low-dose STZ injection

**Figure 3.**
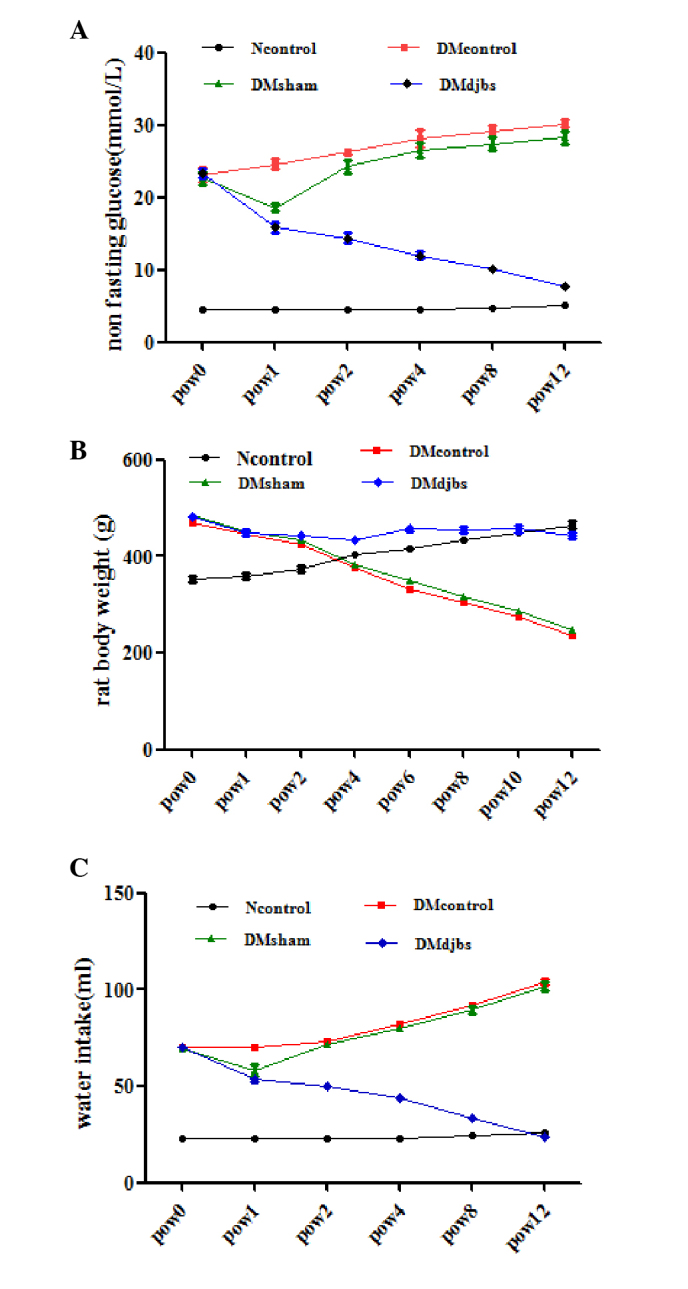
(A) Non-fasting glucose levels of rats in different groups. After DJBS placement for 12 weeks, the glucose level of the DJBS-implanted rats decreased significantly compared with that of the sham surgery and diabetic control rats (F=70.697; P<0.01). (B) Body weights of DJBS-implanted rats decreased slightly, while the body weights of the sham surgery and diabetic control rats decreased significantly compared with those of the DJBS-implanted rats and rats fed a normal diet (F=402.968, P<0.01). (C) Water intake of the DJBS-implanted rats decreased significantly compared with that of the sham surgery and control rats (F=117.19, P<0.01). DJBS, duodenal-jejunal bypass sleeve; Ncontrol, normal control; DMcontrol, diabetes mellitus control; DMsham, diabetes mellitus sham surgery; DMdjbs, diabetes mellitus with DJBS; pow, post-operative week.

On the basis of insulin resistance, rats readily developed overt diabetes mellitus following the injection of a low dose of STZ (35 mg/kg). However, the same dose of STZ did not influence the plasma glucose level of rats fed a normal diet (data not shown). This rat model of diabetes is similar to T2DM in humans. Diabetic rats presented the clinical characteristics of polyphagia, polydipsia (Fig. 3C) and polyuria as compared with the rats fed with a normal diet. The body weights of the diabetic rats continued to decreased throughout the experiment.

#### Effect of DJBS implantation on the remission of diabetes mellitus in rats

At 12 weeks after DJBS implantation, the DJBS was found to have remained intact and in position (Fig. 1D and E). Prior to DJBS implantation, no difference in plasma glucose level was observed among the DJBS-implanted, sham surgery and control diabetic rats (Fig. 3A; 23.3±1.6 vs. 22.7±2.2 vs. 23.1±2.4 mmol/l; P>0.05). At 12 weeks after DJBS implantation, the non-fasting glucose level of the DJBS-implanted rats decreased significantly from 23.3±1.6 to 7.7±0.8 mmol/l, but that of the rats in the sham surgery and diabetic control groups continued to rise (Fig. 3A). The glucose levels of rats in the four groups were significantly different (Fig. 3A; F=70.697; P<0.01) 12 weeks after surgery. The plasma glucose level of the DJBS-implanted rats was significantly lower than that of the sham surgery and diabetic control rats (Fig. 3A; 7.7±0.8 vs. 30.2±1.1 vs. 29.4±1.7 mmol/l; F=862.99; P<0.01). Pairwise comparison showed no difference in plasma glucose level between the diabetic control and sham surgery rats (30.2±1.1 vs. 29.4±1.7 mmol/l; P>0.05). In parallel with the reduction in glucose levels, the symptoms of polyphagia, polydipsia (Fig. 3C) and polyuria were all relieved completely 12 weeks after DJBS placement; however, the symptoms of the sham surgery and diabetic control rats continued to deteriorate (Fig. 3A and C).

Following DJBS implantation, the body weights of the DJBS-implanted diabetic rats decreased only slightly, while the rats in the diabetic control and sham surgery groups continued to lose weight at a greater rate. At pow12, the body weights of the rats in the sham surgery and diabetic control groups were significantly lower than those of the DJBS-implanted and normal-diet-fed control groups (Fig. 3B; 246.5±6.6 vs. 237±12.9 vs. 442.8±14.3 vs. 465±18.3 g; F=402.968; P<0.01.).

#### Effect of DJBS implantation on the plasma and intestinal GLP-1 levels of rats

**Figure 4.**
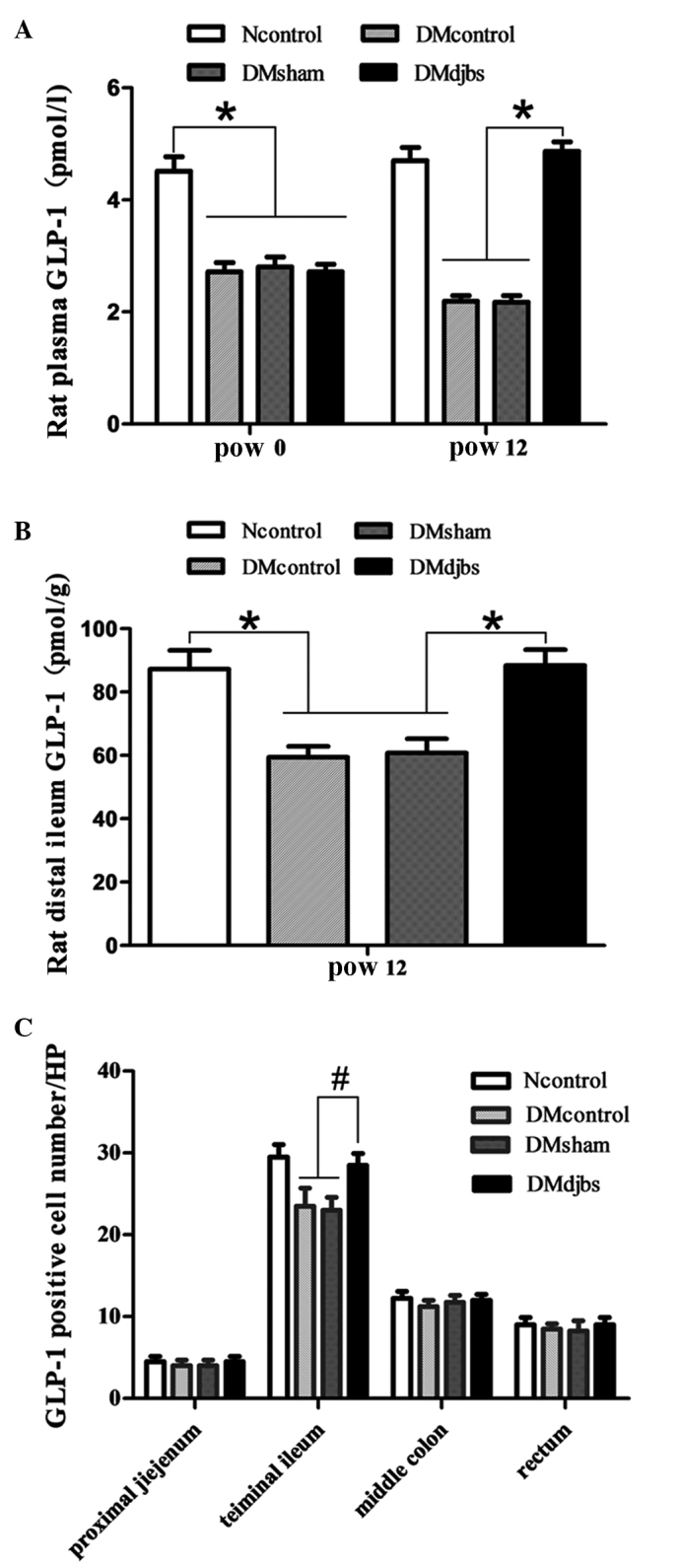
(A) Plasma GLP-1 levels of diabetic rats were lower than those of rats fed a normal diet at pow0 (F=106.37; *P<0.01). After DJBS placement for 12 weeks, the plasma GLP-1 levels of DJBS-implanted rats increased significantly compared with those of diabetic control and sham surgery rats (F=423.237; P<0.01); the GLP-1 levels of the sham surgery and diabetic control rats continued to decrease. (B) The GLP-1 concentration in the distal ileum tissue of DJBS-implanted rats was significantly higher than that of diabetic control and sham surgery rats (F=55.423; *P<0.01). (C) The GLP-1-positive cell number in the distal ileum tissue differed significantly among the four groups (F=5.826; ^#^P<0.05). No significant difference was found in the proximal jejunum (F=0.08; P>0.05), middle colon (F=2.56; P>0,05) or rectum (F=0.56; P>0.05). GLP, glucagon-like peptide; DJBS, duodenal-jejunal bypass sleeve; Ncontrol, normal control; DMcontrol, diabetes mellitus control; DMsham, diabetes mellitus sham surgery; DMdjbs, diabetes mellitus with DJBS; pow, post-operative week.

Prior to DJBS placement, the plasma GLP-1 levels of the diabetic rats declined markedly compared with those of the normal-diet-fed rats following glucose administration. However, at 12 weeks after DJBS placement, the plasma GLP-1 levels of the DJBS-implanted rats were significantly higher than those of the rats in the sham surgery and diabetic control groups (Fig. 4A; 4.87±1.06 vs. 2.17±0.11 vs. 2.19±0.10 pmol/l; F=106.37; P<0.01,). The GLP-1 concentration in the distal ileum of the DJBS-implanted rats was also higher compared with that of the rats in the control and sham surgery groups (Fig. 4B; 88.4±4.9 vs. 60.8±4.4 vs. 59.4±3.5 pmol/g; F=55.423; P<0.01.). However, no significant differences in GLP-1 level were found in tissue from the proximal jejunum, middle colon and rectum. DJBS placement increased the GLP-1 levels of diabetic rats in the plasma and distal ileum tissue.

#### Number of GLP-1-positive cells

**Figure 5.**
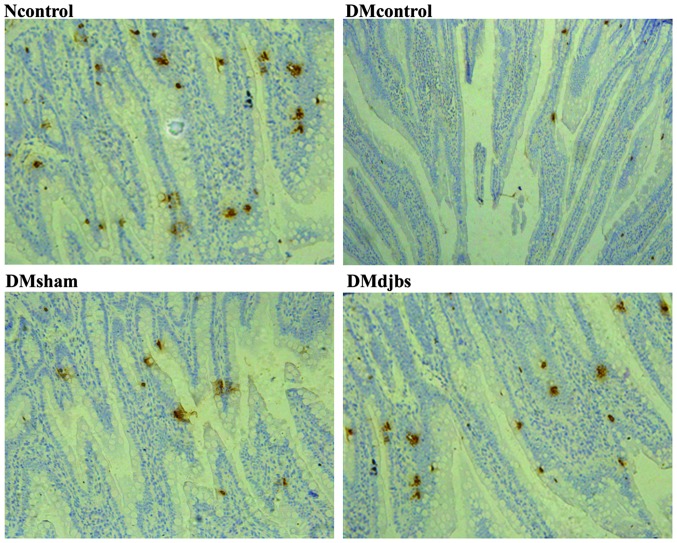
Immunohistochemical staining of GLP-1 in the rat distal ileum in different groups at 12 weeks (magnification, x200). GLP-1, glucagon-like peptide-1; Ncontrol, normal control; DMcontrol, diabetes mellitus control; DMsham, diabetes mellitus sham surgery; DMdjbs, diabetes mellitus with duodenal-jejunal bypass sleeve.

As shown in Fig. 5, GLP-1 was mainly expressed by the intestinal glands. The number of the GLP-1-positive cells increased from the proximal to the distal intestine. When the DJBS implant had been in place for 12 weeks, the GLP-1 positive cell number increased significantly in the distal ileum of the DJBS-implanted diabetic rats (F=5.826; P<0.05). The total number of GLP-1-positive cells in the distal ileum of the Ncontrol, DMcontrol, DMsham and DMdjbs groups was 29.5±3.1, 23.5±4.4, 23.0±3.2 and 31.0±2.6/HP, respectively (Fig. 4C). However, no difference in GLP-1-positive cell number among the groups was found in the tissue from the proximal jejunum, colon and rectum.

## Discussion

GLP-1 is an important gut hormone involved in diabetes. The physiological effects of GLP-1 include stimulation of insulin biosynthesis and secretion, promotion of β-cell proliferation, reduction of food intake and inhibition of glucagon secretion (29,30). Circulating GIP and GLP-1 levels are very low in the fasting state and rapidly increase following food ingestion. In patients with T2DM or impaired glucose tolerance, the plasma GLP-1 level is reduced compared with that in normal individuals (31,32). However, following bypass of the duodenum and jejunum by surgery (33) or luminal sleeve (16), GLP-1 secretion increases again in diabetic patients. This indicates that proximal small intestine bypass can restore impaired GLP-1 secretion in diabetic patients. In the present study, it was also found at that at 12 weeks after DJBS implantation, the glucose-stimulated GLP-1 secretion was greater in the rats with DJBS placement than in diabetic control and sham surgery rats (4.87±1.06 vs. 2.17±0.11 vs. 2.19±0.10 pmol/l; F=106.37; P<0.01; Fig. 3A). The GLP-1 level of the DJBS-implanted diabetic rats increased from 2.72±0.14 prior to surgery to 4.87±0.17 pmol/l, which was comparable with the GLP-1 level in normal rats; however, the rats in the diabetic control and sham surgery groups did not exhibit any improvement. In addition to the increase in plasma GLP-1 levels, the GLP-1 concentration in the distal ileum was also significantly higher than that in the rats of the diabetic control and sham surgery groups (Fig. 3B). Although increases of anorexigenic gut hormones such as GLP-1 have been observed in diabetic patients and obese rats following DJBS implantation (16,26), it is not clear whether increases of circulating GLP-1 levels are associated with enhanced stimulation or proliferation of the enteroendocrine cells or a combination of these factors.

In order to investigate whether the increase in GLP-1 levels after DJBS implantation was associated with an increase in the number of intestinal L cells, changes in the numbers of intestinal GLP-1-positive cells were also observed, and an increase in the number of GLP-1-positive cells in the rat distal ileum mucosa was observed 12 weeks after DJBS placement. An increase in the number of L cells has previously been observed in rats following RYGB surgery (25), since DJBS mimics certain effects of RYGB surgery, and the results of the present study are similar those previously observed in RYGB-implanted rats (25). However, in the present study, an increase in the number of L cells was only found in the distal ileum. This may be because the L-cell density is the highest in the rat distal ileum, and the observation time was not long enough.

The secretion of GLP-1 is influenced greatly by the rate (34) and load (35) of nutrient entry into the small intestine, the flow patterns within it (36), and also the length of small intestine exposed to nutrition (37); GLP-1 secretion is dependent upon >60 cm of the intestine being exposed to glucose (37), and GLP-1 release is prolonged when sucrose reaches the colon (38). Furthermore, the secretion of GLP-1 in response to sucrose is increased when malabsorption is induced by the α-glucosidase inhibitor acarbose (39), which presumably allows stimulation of a greater length or more distal region of the gut by ingested sugar.

Following DJBS implantation, the duodenum and proximal jejunum mucosa are isolated from partially ingested nutrients delivered from the stomach; therefore, the mucosa of the proximal jejunum is directly exposed to partially digested nutrients. The mixing of chyme with bile and pancreatic juice is also delayed until the proximal jejunum. Thus, a direct stimulatory effect of chyme on the duodenal mucosa is absent, but the stimulatory effect of partially digested chyme on the mucosa of the proximal jejunum is improved. The enteroendocrine cells distributed in the digestive tract may detect the improved stimulatory effect of the partially digested alimentary flow and boost hormone secretion. In the present study, it was found that the levels of GLP-1 in the plasma and intestinal tissue increased 12 weeks after DJBS implantation. The increased stimulatory effect of partially digested nutrients on intestinal L cells might lead to the increase in GLP-1 levels in diabetic rats after DJBS implantation.

Following the placement of a DJBS in the duodenum and proximal jejunum, interaction between chyme, bile and pancreatic juice is avoided. Also the absorption of nutrients is delayed to the jejunum below the end of the DJBS; thus, partially digested nutrients will arrive at the small intestine at greater concentrations and contact the intestinal mucosa for a longer distance. The intestinal mucosa will, therefore, interact with greater quantities of incompletely digested nutrients, and the increased stimulatory effect on L cells may lead to an increase in GLP-1 secretion. Since L cells produce both GLP-1 and GLP-2, and GLP-2 can promote growth of the intestinal mucosa, it is possible that the more strongly stimulated L cells also release more GLP-2, which will stimulate the growth of the intestinal mucosa. Thus, hypertrophy of the intestinal mucosa will increase the total number of L cells. Hypertrophy-dependent doubling of L cells has been observed in rats following Roux-en-Y gastric bypass surgery (25); however, in the present study, the GLP-1-positive cell number was found to increase only in the rat distal ileum after DJBS placement for 12 weeks. This is probably due to the observation time not being long enough and the L-cell density being highest in the rat distal ileum. Whether the increased number of GLP-1 positive cells is associated with hypertrophy of the intestinal mucosa or L-cell proliferation requires further investigation.

In the present study, only the change in the number of L cells and the association with plasma GLP-1 level were investigated; the expression of GLP-1 at the genetic level was not tested. However, the GLP-1 level was detected by ELISA in intestinal tissues and blood. An ELISA is able to determine GLP-1 concentration quantitatively. The increased blood GLP-1 level might be attributed to the increased L-cell number in the distal ileum.

In summary, DJBS placement can effectively induce glycemic control in rats with diabetes induced by a high-fat diet and low-dose STZ injection; 12 weeks after DJBS implantation, the diabetes mellitus was relieved completely. Concurrently, the GLP-1 level and intestinal L-cell number also increased markedly compared with those in the sham surgery and control groups. DJBS implantation-induced changes to alimentary nutritional digestion and absorption may enhance the stimulation of L cells, thereby boosting GLP-1 secretion and contributing to the DJBS-induced remission of diabetes in rats. However, the specific molecular entities that stimulate the L cells and the exact pathway involved in boosting the GLP-1 release require further investigation.
